# Ionomer-Based Ion-Sensitive Field-Effect Transistor for Lithium Ion Sensing

**DOI:** 10.1021/acsomega.5c05196

**Published:** 2025-11-26

**Authors:** Tuluhan Olcayto Colak, Mehmet Kurt, Ecenaz Yaman, Nurdan Demirci Sankir, Mehmet Sankir

**Affiliations:** † Micro and Nanotechnology Graduate Program, 52995 TOBB University of Economics and Technology, Sogutozu Caddesi No 43 Sogutozu 06560 Ankara, Turkey; ‡ Department of Materials Science and Nanotechnology Engineering, TOBB University of Economics and Technology, Sogutozu Caddesi No 43 Sogutozu 06560 Ankara, Turkey

## Abstract

Lithium detection is critical in the medical field, as it is used for the treatment of bipolar disorder. Here, we offer a simple method using an ion-sensitive field-effect transistor device prepared to detect the presence of lithium in a solution. The Nafion 115 membrane used in this study has been conditioned to allow the transport of lithium ions from the electrolyte to a zinc oxide-based transistor. The device was studied with lithium ion concentrations ranging between 10^–2^–10^–7^ M. It was modeled using the COMSOL Multiphysics program for 10^–2^ M. The device showed 2.85 mA/decade sensitivity and a detection limit of 77 μM.

## Graphical abstract

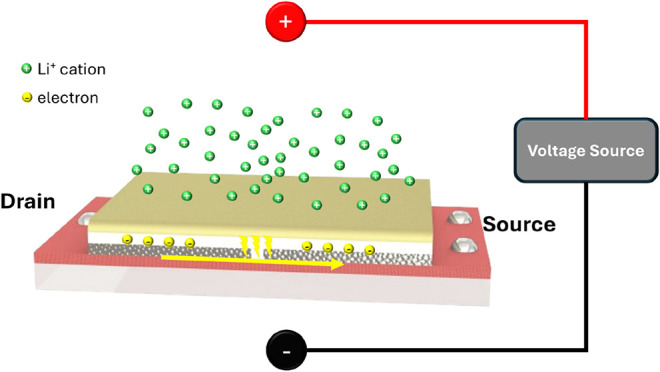

## Introduction

Through its mood-stabilizing properties, lithium is the main treatment for bipolar disorder^1,2^ and other health problems^3,4^ and requires regular checking.^5^ There are a great number of studies throughout the literature on the detection of lithium ions (Table S1). Singh and Kumbhat^6^ presented an easy fabrication and sensing approach for an electrochemical sensor strip that was functionalized with a 14-crown-4 ether-based ionophore. Another study was conducted by Gupta et al.,^7^ in which they developed a carbosiloxane dendrimer for the purpose of detecting lithium ions. Obare and Murphy^8^ have developed a lithium-selective variation of the dipyridophenazine (DPPZ) ligand, when bound to lithium ions, changes the color of its emission. Furthermore, thin films of LiMn_2_O_4_ have proven selective lithium detection by showing Li^+^ intercalation from aqueous solutions is both quick and reversible.^9^ Teixeira et al.^10^ investigated a graphite–epoxy electrode which was working within a molar range of 10^–6^ to 3.3 × 10^–2^.

Here, we present a system including an ionomer-based lithium-ion transporter and a nonenzymatic ISFET sensor built on an FTO substrate, using ZnO as a channel and Nafion 115 as a gate. The functionality of the device depends on the Nafion 115 cation exchange ionomer membrane, which serves as the Li^+^ transporter in ISFET systems and sensors during linear sweep measurements. Ionomer membranes, particularly perfluorosulfonic acid variants such as Nafion, may become crucial in ion-sensitive field-effect transistor (ISFET) applications due to their unique properties, including ionic conductivity, chemical stability, selective ion permeability, and the ability to serve as a supportive matrix or protective barrier. ZnO attracts attention due to its eco-friendly and nontoxic properties, as it can be produced in a controlled manner with easy and cost-effective methods of thin films for various applications.^11–14^ It has excellent electrical properties and has demonstrated significant potential in the development of various ZnO-based sensors and sensing platforms.^15–17^ An advantage of these films is the ability to modify their properties by introducing a donor or acceptor impurity.^18–23^ ZnO-based ISFET devices have been mostly used for pH sensing.^17,24–26^ However, a device in which ZnO and ionomer membranes are used together and lithium ions are determined, as in this study, has not been reported previously. Previously, all-solid-state two-electrode photosupercapacitors constructed by our group have used lithiated Nafion membranes as separators.^27^ There are quite a few studies on cation determination in the literature.^28–31^ However, the most fundamental disadvantages of these studies are that they either perform quantitative determination with enzymatic methods^32–36^ or, in the case of nonenzymatic methods, the material to be determined goes through multistage and complicated production processes. For example, Singh and Kumbhat^6^ have used a gold disc as the substrate of their electrode, on which they have grown crown ether structures as ionophores with repeated incubation times, adsorption steps.

This study proposes a method that makes use of the Nafion 115 membrane that is manufactured in large quantities presently and uses it with a ZnO semiconductor grown via sputtering on a fluorine-doped tin oxide glass, which is also produced at a mass scale already. Also, using COMSOL Multiphysics, the modeling of the ISFET device was studied with nonstandard ISFET geometry and scale, and current outputs were examined by scanning under different fixed potentials. With this study, ideal conditions and materials for the prepared ISFET devices can be optimized. By modeling the operation of ISFETs, we aimed to find out if a polymeric cation exchange membrane can be modeled to emulate the device’s current response under bias potential.

## Experimental Section

### Preparation of Device

Fluorine-doped tin oxide (FTO) substrates were cleaned in four steps in an ultrasonic bath containing an Alconox solution, deionized water, ethanol, and acetone, respectively. After the cleaning process, the substrates were placed in an oven at 60 °C and dried. The titanium layer was deposited on substrates via sputtering, using a Ti target (99.99% purity, Plazmaterials) and a Vaksis Midas PVD-MT/2M2T sputtering device. During the coating process, the chamber where the coating will be performed is first brought to a pressure of 7.6 × 10^–6^ Torr, and the coating is carried out by sending high-purity (99.99%) argon gas to the environment. The plasma discharge was produced by continuously supplying 100 W for 16 min. After sputtering, a 1064 nm wavelength ytterbium fiber laser (FiberLAST) was used for the selective ablation of the Ti layer. An area of 100 μm thickness and 15 mm length was drawn over a Ti-coated FTO substrate at 15 W power, 70 kHz impulse frequency, and 2200 mm·s^–1^ step time, and both Ti and FTO over that area were removed.^37^ After the FTO layer removal, the sample was taken to the RF magnetron sputtering system again for ZnO deposition, and the ZnO layer deposition process was carried out by continuously applying 60 W plasma discharge for 9 min under 7.6 × 10^–6^ Torr. After the coatings were made, the contact connections were prepared with insulated copper wires and silver epoxy. The contacts were covered with insulation of epoxy adhesive for protection. Figure 1a–e summarizes the preparation stages of the transistor. The schematic representation of the prepared ISFET is given in Figure 1f.

**Figure 1:**
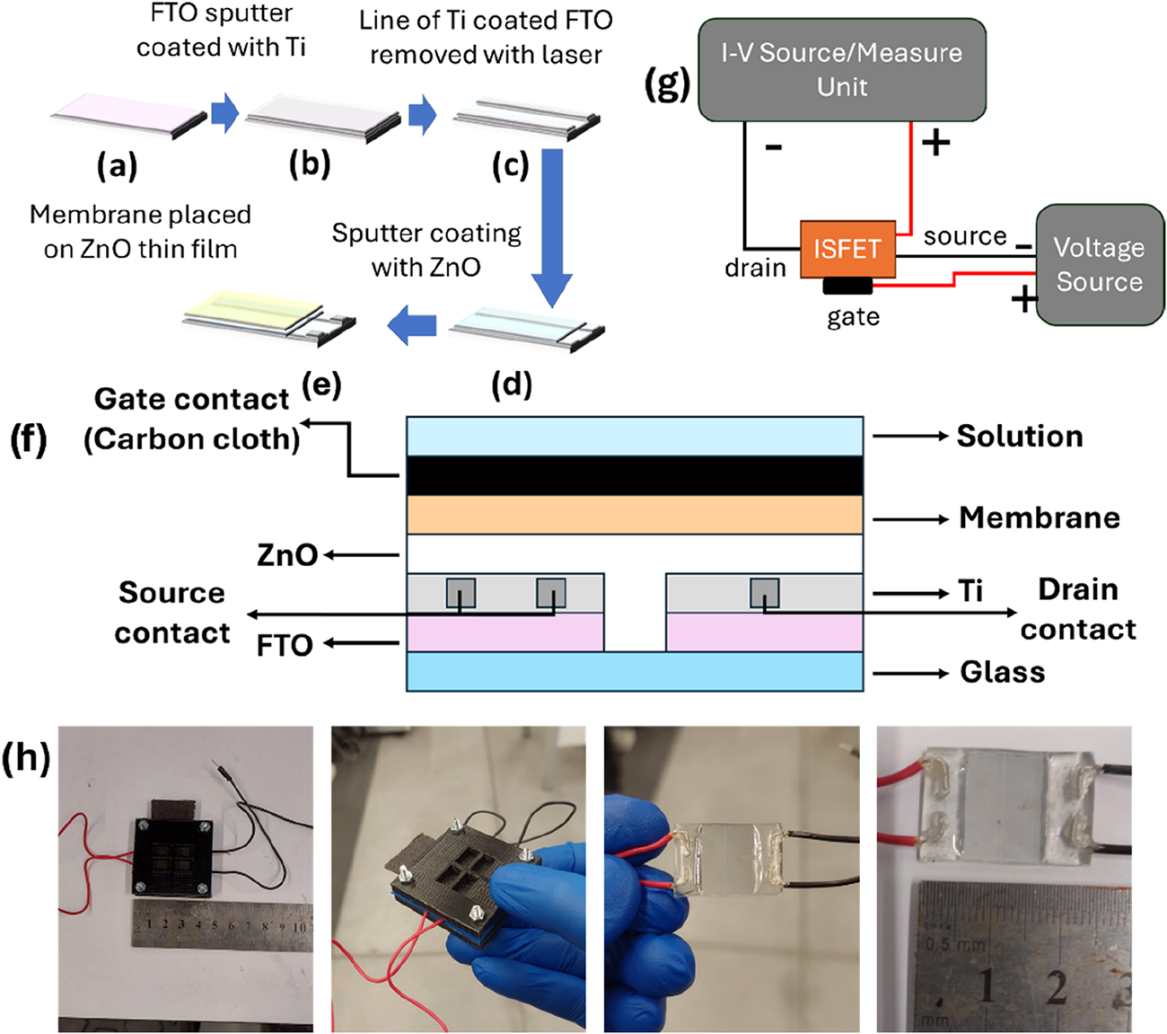
ISFET design and building method: The FTO-coated glass was the substrate (a), FTO coated with Ti (b), then a 100 μm wide strip of Ti and FTO layer was removed with a YAG laser (c), ZnO layer was sputter-coated on this coating (d), finally, Nafion 115 membrane was placed on top of this structure (e). Cross-section of the final device (f). Circuit connections of the ISFET for measurements (g). Images of the final device (h).

### Preparation of Membrane

To sense lithium ions, Nafion membranes must be conditioned first. To do so, Nafion 115 membranes were boiled in 500 mL of 0.5 M sulfuric acid for 2 h and afterward another 2 h in deionized water. This process cleans the membrane surface, hydrates it, and allows for transport of protons through the membrane. After the initial conditioning, membranes were allowed to rest for a night. Afterward, conditioned Nafion 115 membranes were boiled for 2 h in 250 mL of 1 M lithium chloride solution in a crystallizer. During boiling, membranes were kept inside the solution, preventing floating, to ensure equal and homogeneous introduction of lithium ions into the membrane. After boiling in lithium chloride solution for 2 h, the membranes were boiled for another 2 h in deionized water. When this process was completed, the membranes were placed in a beaker and rested in deionized water overnight.

Solartron 1260 and 1278A electrochemical interfaces were used for the conductivity measurements of the prepared membranes. Measurements were made at 25 °C using the Bekktech cell with the 4-electrode method on the membranes. Figure S1a,b shows the electro impedance spectroscopy result taken after the initial and second conditionings were complete, respectively. The membrane displayed a proton conductivity of 0.1 S·cm^–1^, which is denoted as the proton form. After the second conditioning, the membrane loses its proton conductivity significantly, which has dropped to 0.013 S·cm^–1^. This is due to the protons present in the sulfonated regions of the Nafion, which were exchanged with lithium ions.

Within the scope of the study, to make the system more practical, the measurement method and the transistor gate were changed from the common method used in the literature. Carbon fabric was used instead of the Ag/AgCl reference electrode to make a connection between the source contact and the gate. For this study, the cell was printed from a 3D printer using PLA+. Carbon fabric was placed on the membrane (Figure 1f).

The source was connected to the positive electrode of the Keithley 2400 (*I*–*V* source/measurement unit), and the drain was connected to the negative end of the same device. The other contact of the source is connected to the negative end of the constant voltage source, and the gate contact is connected to the positive end of the constant voltage source. Measurement configuration was to take measurements by applying a potential between the source and the gate, with the negative (−) end of the potential applied from the voltage source connected to the source electrode and the positive (+) end to the gate electrode and by connecting the contacts coming from the source and the drain to the Keithley 2400 device (Figure 1g). After connections were prepared (Figure 1h), the study with lithium chloride was tested at 100 nM, 1 μM, 10 μM, 0.1 mM, 1 mM, and 10 mM concentrations under 0 V, 1, 1.5, and 2 V gate potential (Figure 2). The electrolyte solutions used during the measurements were dropped onto this electrode. What is expected in the circuit is that the current output of the ISFET increases depending on the increase in electrolyte concentration. In the measurements taken, it was observed that the currents between the voltage differences were in the μA - mA range. The measured drain current increased, depending on the increasing source-gate potential and in direct proportion to sweep potential. The increase in the amount of positively charged particles in the electrolyte allows more charge to pass through the ion-permeable membrane, causing the zinc oxide semiconductor layer to accumulate more charge carriers, and thus the amount of current passing through the circuit increases. The positively charged cations in the electrolyte fill the channels in the membrane, move through the structure, and create an electric field on the semiconductor. This is achieved by the resistance of the membrane to ion movement and the formation of a capacitance between the cation concentrations in the carbon fabric and the zinc oxide layer. Since this capacitance is directly proportional to the number of particles, it will increase the current to pass through the circuit. The increase in the cation concentration on the carbon fabric allows the ISFET circuit to give a higher current.

**Figure 2:**
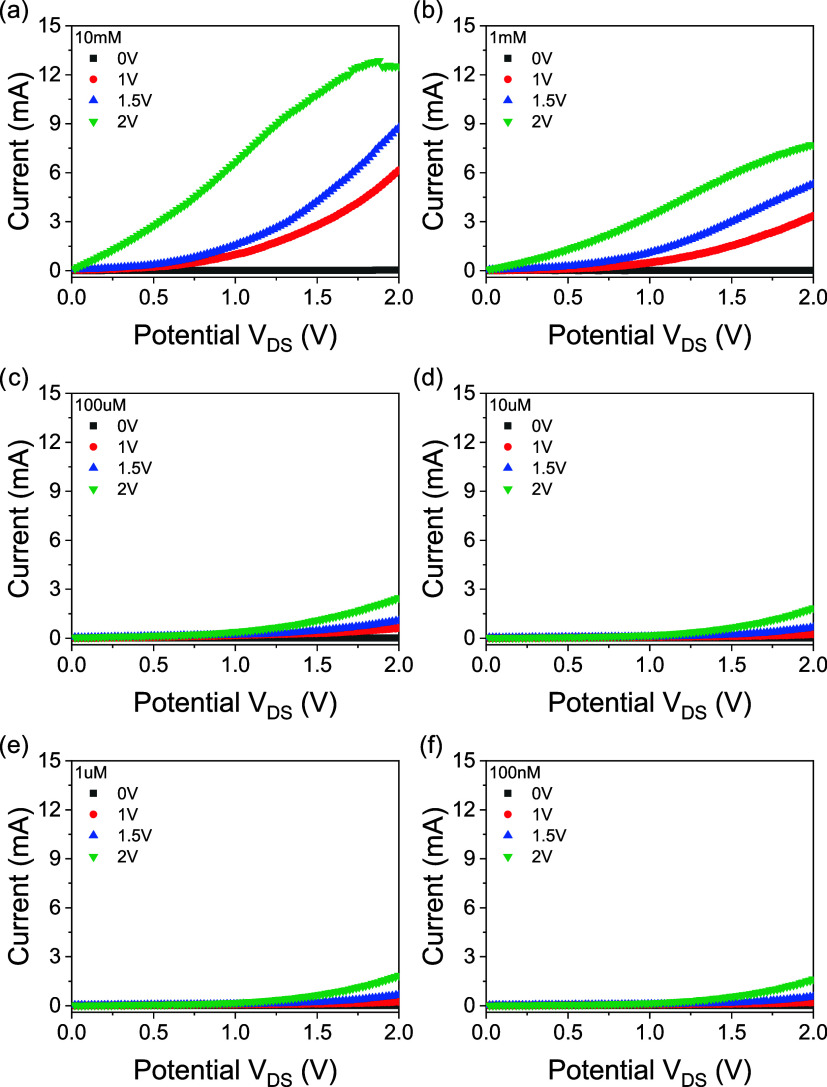
Linear sweep of ISFET device between 0 and 2 V, showing results for concentrations of 10 mM (a), 1 mM (b), 100 μM (c), 10 μM (d), 1 μM (e), and 100 nM (f). Measurements were taken at gate potentials of 0, 1, 1.5, and 2 V.

## Results and Discussion

The produced membranes’ conductivities were measured at 25 °C using the Bekktech cell using the 4-electrode method of the membranes, with the use of Solartron 1260 and 1278A electrochemical interfaces. Under the condition of constant gate-source potential, the results of the linear source-drain potential sweep for the conditioned membrane are displayed in Figure 2.

For concentrations of 10 mM, 1 mM, 100 μM, 10 μM, 1 μM, and 100 nM, the gate potentials were 0, 1, 1.5, and 2 V and each concentration was tried with each gate potential. Based on these findings, it was determined that the potential sweep between the source and the drain exhibited variations based on the ion concentration that was present in the solution that was dropped on the device. As the concentration of the solution increases, the slope of the *I*–*V* curve also gradually increases. Another observation that was made was that the slopes of the *I*–*V* curves increased with increasing gate potential when the concentration of lithium ions remained constant. A direct correlation exists between this behavior and the typical behavior of the transistors. We have displayed the change in current depending on lithium concentration under constant gate voltage (Figure 3a) and the change in current depending on gate potential under constant solution concentration (Figure 3b) using the drain current values that were acquired from the *I*–*V* curves that are presented in Figure 2. Electro impedance spectroscopy results (Figure S2) were used to calculate intrinsic conductivity. The resulting plot for conductivity calculation has been taken for concentrations of 10, 20, 40, 60, 80, and 100 mM and is given in Figure 3c. From calculations, the intrinsic conductivity of the Li^+^ form of Nafion 115 was determined to be 0.86 S. cm^–1^ at 25 °C. Upon examination of both plots, it was discovered that the ISFET device does not produce any charge when the gate potential is set to 0 V. When the voltage at the gate is 1 V, the device begins to react to the presence of ions on its sensing layer, and the drain current will increase in tandem with the gate voltage. Calculations were made to determine the sensitivity values based on the differences in concentration and drain current in order to determine the operating potential and concentration limitations of the device. These values are presented in Table 1, Table S2. Figures S3 and 3d illustrate how they have changed in relation to the changes in concentration. Moreover, the effect of gate voltage, ranging between 0 and 2.5 V, on the source-drain current–voltage response was investigated at 100 μM LiCl concentration (Figure S4).

**Figure 3:**
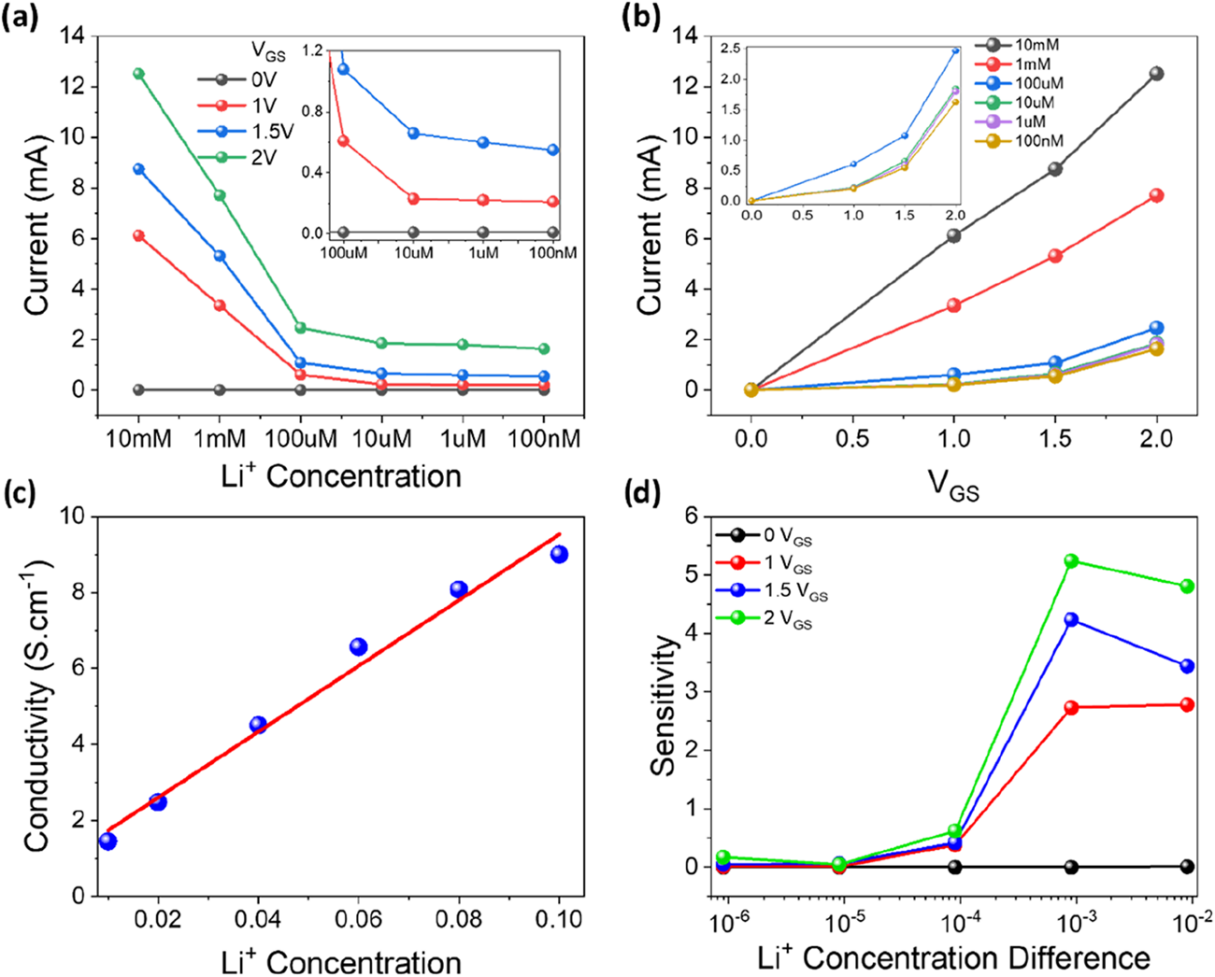
Plot of concentration vs current for each gate potential applied (a) and gate voltage vs current for each LiCl concentration (b). Conductivity calculations of Li^+^ form Nafion 115 in various lithium chloride solutions (c). Plot of sensitivity values from Table S2 (d).

**Table 1:** Linear Fitting Calculations from the Calibration Curve of the Device Show the Sensitivity under Different Bias Potentials

plot	1 V	1.5 V	2 V
intercept	9.715 ± 1.51693	13.925 ± 2.12284	19.175 ± 2.76525
slope	2.04 ± 0.41286	2.85 ± 0.57776	3.725 ± 0.75261
R-square (COD)	0.92429	0.92405	0.92452
Adj. R-square	0.88643	0.88607	0.88678

1 $$S={\frac{\left|\partial I_{D}\right|}{\left|\partial \mathrm{pH}\right|}}_{V_{\mathrm{GS}}\left(\mathrm{constant}\right)}$$

Calculated for *V*_GS_ values of 0, 1, 1.5, and 2 V, eq 1 can be used to express ISFET sensitivity. S is defined as the absolute value of the change in *I*_D_ per decade change in concentration, with fixed *V*_GS_. Once the drain current *I*_D_ is calculated for a certain gate voltage at a certain concentration value, *I*_D_ can be found again for a neighboring concentration value.^38^ To get sensitivity, linear fittings of the calibration curve were taken in order to calculate the change in current depending on electrolyte concentration under constant gate bias. The data calculated from Figure S3 is given in Table 1. The device prepared has a sensitivity value of 2.85 mA/decade at 1.5 V gate bias potential and a detection limit of 77 μM.

From Table 1 and Figure 3d, it can be seen that the device works best at 1.5 V gate potential, and it can differentiate between 100 nm and 1 μM because it shows the most linear change of sensitivity at lower concentration values. We can compare our results in Table 1 to examples from the literature in Table 2. It can be seen that the result of the device prepared with a Nafion 115 cation exchange membrane shows comparable sensitivity but a higher detection limit.

**Table 2:** Literature Examples of Devices Prepared with Nafion Membranes

device	detecting ion	limit of detection	sensitivity	references
Reference FET			52.1 mV/pH	^41^
Graphene field-effect transistor	RNA	1 fM		^42^
Solution-gated transistor	glucose	0.5 μM	173 mV/decade	^43^
Solution-gated transistor	dopamine	10 nM	188 mV/decade	^44^
Reference FET	H^+^		5.8 mV/pH	^45^
ISFET	ascorbic acid	0.01 mM		^46^
Organic electrochemical transistor	epinephrine	0.1 nM	533 mV/dec	^47^
ENFET	glucose	0.02 mM		^47^
ENFET	glucose	0.025 mM	48 mV/pH	^48^
pH sensing electrode	H^+^		56.6 mV/pH	^49^
Solid state reference rlectrode			57.5 mV/pH	^50^
ISFET	Ca^2+^		35 mV/decade	^51^
pH EG-FET sensor	H^+^		91.1 mV/decade	^52^
Urea sensor	Urea		57.7 mV/pH	^53^
OECT	H_2_O_2_	30 nM	147 mV/decade	^54^
OECT	H_2_O_2_	300 nM	1.5 mA/decade	^55^
ISFET	Li^+^	77 μM	2.85 mA/decade	this work

The ability of the membranes to transfer ions across their distinct medium is the source of the ability to discern between each concentration. Additionally, the conditioning of the membrane makes it easier for particular ions to attach to its channel structures, which, in turn, makes it possible for those ions to move through the membrane. For the sake of this illustration, lithium ions are attached to the membrane. The passage of ions through the solution is made possible by the ions that are connected to the membrane. According to this method, the simple presence of ions above the ISFET device is not sufficient to produce a charged layer, which is required for the device to commence electron transport from the source to the drain. In order to put this concept to the test, an ISFET device that utilized a PTFE gate rather than a Nafion 115 gate was studied. While the Nafion 115 membrane has a thickness of 127 μm, the PTFE membrane has a uniform thickness of 140 μm that is consistent throughout. On the other hand, Nafion 115 is capable of carrying a charge and transporting ions, whereas PTFE does not possess this characteristic. The effect of ion transport through the body of the membrane might be demonstrated by a sweep voltammetry experiment, provided that the thicknesses of the membranes are comparable. It was determined that a potential sweep was carried out for a 1 mM LiCl solution under gate potentials of 0, 1, 1.5, and 2 V. It was noticed that there was no charge being carried across the ISFET device with the PTFE gate (Figure S5) when the measurement was taken from the device. Clearly, this demonstrates that Nafion 115 does, in fact, enhance the transfer of charge and makes it possible for ISFET devices to react to varying concentrations. The effect of temperature was also observed for the ISFET device (Figure 4a) to further the investigation. The transistor was placed in a water bath with a container filled with 100 μM LiCl. The temperature was gradually increased to 20, 30, and 40 °C. 1.5 *V*_GS_ gate bias was set, and *I*_DS_ for each of them was recorded as 23, 27, and 28 mA for 20, 30, and 40 °C, respectively. It is noted that the device increased its drain current 17% when heated from 20 to 30 °C; however, it was noted that this increase was only 3% when the temperature was increased from 30 to 40 °C.

**Figure 4:**
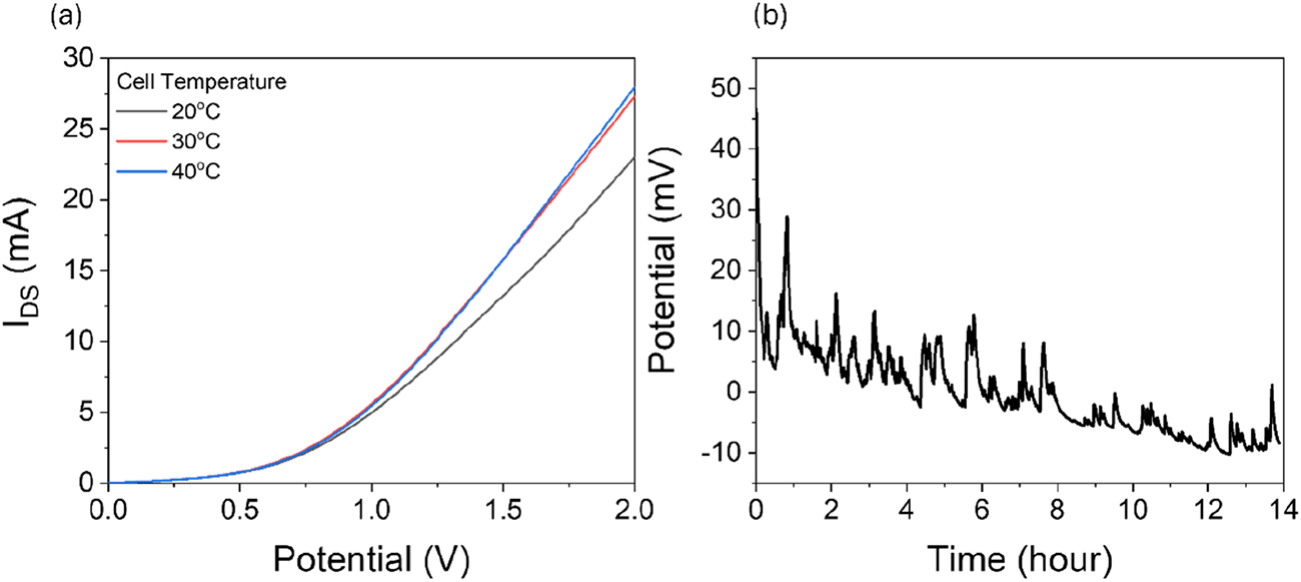
Current response of the designed ISFET device under the effect of increasing temperature (a). The 14 h open-circuit potential of the device while submerged in 0.1 M LiCl solution (b).

To observe the drift occurring on the transistor, the device was placed in a 0.1 M LiCl solution, and open open-circuit potential was recorded for 14 h. In Figure 4b, it can be observed that there is significant drift in the open-circuit potential of the device. The rate of drift was 2.85 mV/hour. There are studies available in the literature to remedy this. Goel et al.^39^ reported a 55% reduction in the drift rate of CMOS ISFET arrays by employing monolayer graphene sheets deposited onto the passivation layers of the ISFET. The decrease in drift rate results from the ion-impermeable graphene layer, which diminishes the chemical alteration of the passivation layers. Therefore, graphene must offer physisorption sites for ions in the electrolyte, ensuring pH sensitivity for ISFET sensors. Pantelli et al.,^40^ postprocessed CMOS ISFET pH sensors by manually applying monolayer and multilayer graphene sheets onto the sensing membrane via an in-house fabrication technique. Graphene serves as a barrier against ion penetration in passivation layers and offers physisorption sites essential for the creation of an electrical double layer, contributing to the pH sensitivity. Real-time drift studies demonstrated, and a two-exponential modified dispersion model corroborated, that graphene decreases the drift in CMOS ISFETs by 50%. The applications of this methodology pertain to the forthcoming generation of ISFET sensors, and further research may yield viable methods to address drift at its source.

Finally, in the experimental part, the time dependence of the *I*_DS_ current output was investigated (Figure 5). Four concentrations with the highest current output were selected. Devices were submerged in 10 μM, 100 μM, 1 mM, and 10 mM LiCl electrolytes.

**Figure 5:**
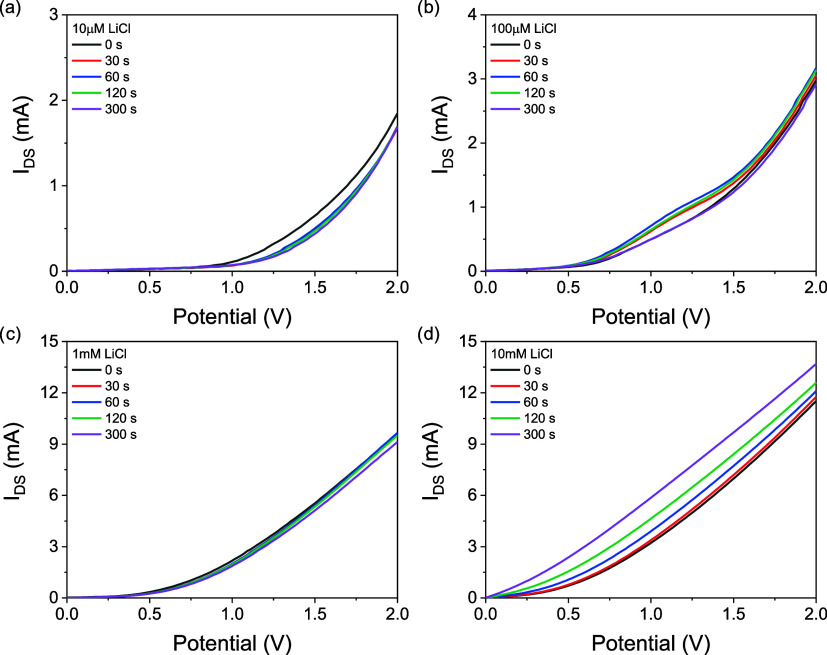
Change in *I*_DS_ output of ISFET device changing with time for devices prepared with 10 μM (a), 100 μM (b), 1 mM (c), and 10 mM (d) LiCl electrolytes.

Usual response for 10 μM, 100 μM, and 1 mM was that after 30 s the device stabilizes, and after 300 s, the current response drops slightly but still levels. It is possible that the stabilization of the current output from these concentrations was related to the equilibrium of the membrane. However, when 10 mM LiCl was used as the electrolyte, there was a gradual and quite visible increase in current output. In a previous study,^56^ we have shown that by merely soaking a sulfonated cation exchange membrane, it is possible to exchange the protons from its initial conditioning with the desired cation. The cation in that study was also a lithium ion. So, a possible reason for this is that the amount of cations present is high enough to pass on even without encouragement from the gate bias potential and increase the drain current. To eliminate this effect, the 30s data have been selected for further device fabrication.

Nafion membranes exhibit chemical and physical stability due to their composition of perfluorosulfonic acid (PFSA), which remains inert under oxidative or reductive circumstances. They are utilized in PEMFCs and in several electrochemical applications. Their lifespan may be influenced by temperature, humidity, or generation of radicals. These membranes can be optimized for prolonged functionality in both acidic and basic environments.^57–61^ Modified PFSA membranes can operate at temperatures up to 150 °C and pressures of 5 atm.^61^ During the evaluation of water electrolyzers, PFSA membranes can demonstrate stable performance during 10,000 h of testing.^62^ Nafion can operate in a fuel cell for over 60,000 h, offering an exceptional longevity.^63,64^ Research indicates that Nafion 115 possesses an extended shelf life attributable to its intrinsic chemical resistance, perhaps lasting indefinitely when stored properly away from pollutants, but slight oxidation may occur over the years without compromising effectiveness. The operational life of Nafion 115 in electrolyzers can attain commercially significant durations.

Cyclic voltammetry of the device was taken with several solutions (100, 10, 1, 0.1, and 0 mM) (Figure S6), and a capacitance of 29 μF was observed to be decreasing steadily with lowered lithium cation concentration. The capacitance was generated between the electrolyte and ZnO layer by the Nafion membrane. The membrane could generate capacitance by keeping charges separate. With increased capacitance, there are more negative charges gathered closer to the surface of the ZnO thin film, which increases the conductivity by lowering resistance.

In Figure S7, a comparison of the device prepared with 100 μM LiCl solutions and 100 μM LiCl plus an equimolar mixture of Na^+^, K^+^, and Ca^2+^ cations was dropped onto the device. A small decrease in current was observed. This was expected as the membrane’s conditioning would not allow a heightened presence of ions outside the recipe to generate current.

### Modeling of the ISFET Device

In this part of the study, the electrical response of the ISFET structure was analyzed based on the current values applied to the source and drain regions within the COMSOL Multiphysics simulation. Modeling parameters are given in Tables S3–S6. The modeling geometry was built following the design given in Figure 6.

**Figure 6:**
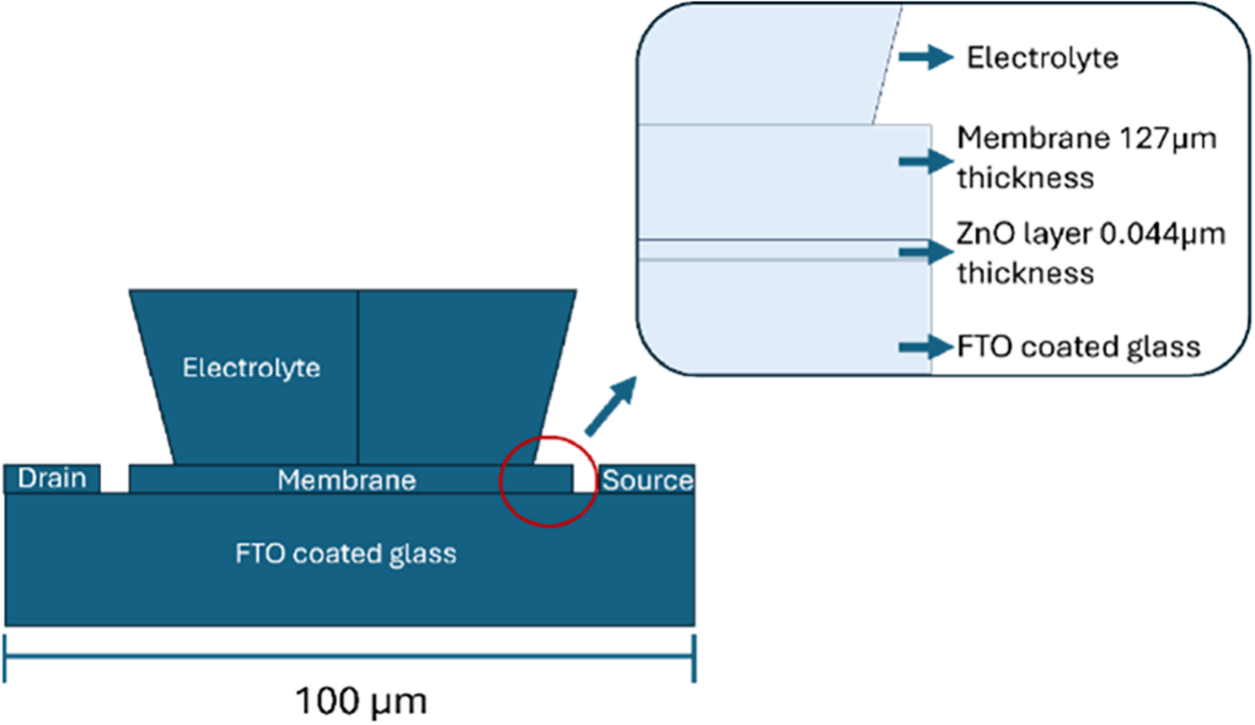
Geometry and scales of the ISFET device used for COMSOL modeling. The inset graph shows the layers of the device.

Figure 7a,b shows the results for the modeling of experimental results for a 10 mM LiCl concentration. The curve in Figure 7a,b illustrates the relationship between the output current of the Li^+^ sensor and the Li^+^ concentration in the solution for two different gate voltages (*V*_G_ = 1.5 V and 1.0 V). These graphs illustrate the sensor’s response to variations in gate potential. This indicates that the modeled sensor is responsive to lithium with comparable behavior to the experimental ISFET device. It is observed that the model is compatible with the experimental study, especially at low *V*_G_ values. In Figure 7c, the spatial fluctuation of the electrolyte potential (*V*) of a 2D-ISFET with respect to the y-position (μm) is examined. The dotted curves denote the one-dimensional method, whereas the solid curves signify two-dimensional modeling. The utilization of the Nafion 115 membrane as the gate material markedly alters the potential distribution at the electrolyte-gate interface. Nafion 115 is an ion exchange membrane characterized by high ion conductivity, which interacts with the surface charge density upon contact with the electrolyte, hence influencing the potential distribution. The graph illustrates that the electrolyte potential varies logarithmically along the *y*-axis, exhibiting a pronounced increase as it nears the surface. Nafion 115 induces the redistribution of charge carriers and the establishment of an electrical double layer, owing to its elevated ionic conductivity. The distinctions between the 1D and 2D models illustrate the variations in geometric effects and electric field distributions influenced by the gate effect. The use of Nafion 115 reveals variations in the width and slope of the potential gradient zone, attributable to the membrane’s ionic charge transport characteristics. This analysis is essential for comprehending and enhancing the electrode-gate interactions of ISFET-based sensors. Figure 7d illustrates the electric potential distribution observed under the influence of the LiCl electrolyte and the Nafion 115 membrane used in our COMSOL-based ISFET model. Analyzing the simulation results presented in Figure 7d reveals that the potential gradient within the electrolyte region is relatively low, while the electric potential is predominantly concentrated around the gate region. The Nafion 115 membrane, with its high ionic conductivity, effectively transmits the surface potential, and the Li^+^ ions contribute to the formation of a potential at the gate–electrolyte interface. Among the key parameters considered in our ISFET modeling, the relative permittivity of the LiCl electrolyte solution used is particularly significant. A decrease in the electrolyte’s relative permittivity reduces the medium’s ability to screen electrostatic fields, thereby enhancing electrostatic interactions and shortening the Debye length. Consequently, this leads to a reduction in the double-layer capacitance. As a result, the transmission of the surface potential to the semiconductor channel is weakened, and a general decrease in current is observed in the *I*–*V* characteristics as the relative permittivity decreases. In our model, an increase in Nafion thickness leads to a reduction in the capacitance between the gate and the semiconductor channel, thereby weakening the influence of the applied gate voltage on the channel. As a result, an increase in gate oxide thickness is associated with a decrease in current levels observed in the *I*–*V* characteristics. The key material parameters of ZnO presented in the table generally influence the increase or decrease in current. Among these parameters, electron mobility is the most critical factor contributing to the increase in current, as higher mobility enables charge carriers to move more efficiently and rapidly through the channel, thereby enhancing conductivity. On the other hand, the band gap is one of the primary factors responsible for the decrease in current. A wider band gap reduces the number of thermally generated free carriers, leading to lower conductivity within the channel.

**Figure 7:**
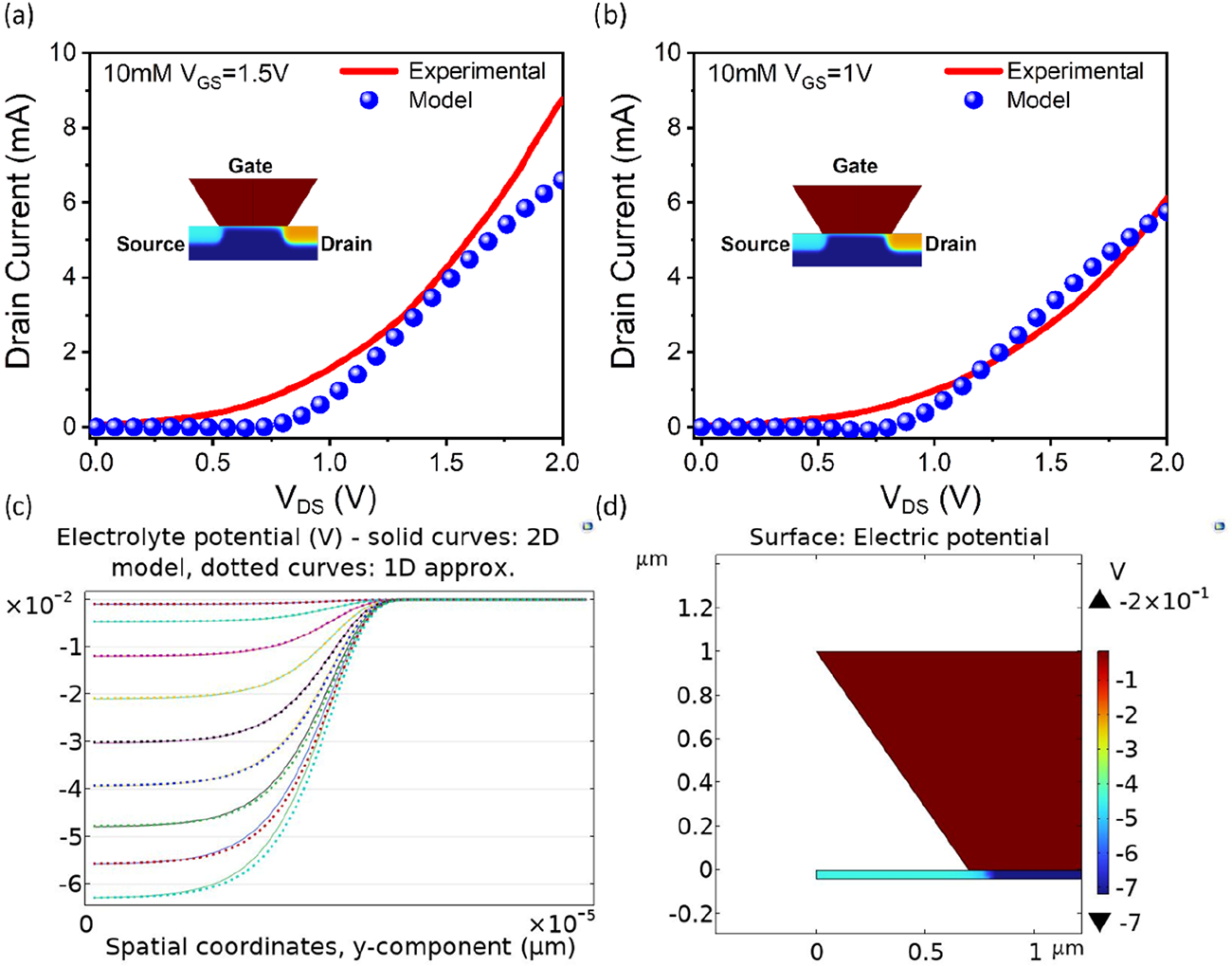
Comparisons of experimental linear potential sweeps with simulated results for a concentration of 10 mM at 1.5 V (a) and 1 V (b) gate potentials. Electrolyte potential near the stern layer along the centerline at different Li^+^ (c). The proposed geometry and electric potential of the simulated ISFET device (d).

Experimental results indicate that the variation in the molarity of LiCl is a significant parameter for *I*–*V* characteristics and curves. In both experimental studies and simulations, an increase in the concentration of the LiCl electrolyte solution leads to an increase in the current in the *I*–*V* plots. This effect is attributed to the enhanced conductivity of the electrolyte and the alteration of the surface electrochemistry of the ISFET. Moreover, the increase in LiCl concentration compresses the electrical double layer (EDL) at the ISFET surface, thereby improving the channel conductivity and shifting the threshold voltage.

## Conclusion

An ion-sensitive field-effect transistor device sensitized to lithium in a solution is used here. Ion-sensitive field-effect devices can use polymeric cation exchange membranes instead of silicon dioxide gates to select and transport ions. Nafion 115 membrane was utilized to transfer lithium ions from an electrolyte to a ZnO-based transistor and detect concentrations between 10^–2^ and 10^–5^ M. The membrane’s conductivity was also determined to be 0.86 S.cm^–1^. The device showed 2.85 mA/decade sensitivity and a detection limit of 77 μM. COMSOL Multiphysics simulations were run using Nafion 115 to investigate ISFET lithium sensing capability. We modeled ISFETs to see if a polymeric cation exchange membrane could match the current response of the device at 10 mM LiCl concentration.

## Supplementary Material



## Notes

Experimental results of electro impedance spectroscopy, *I*–*V* sweep curves and cyclic voltammetry, and tables for model parameters (PDF)
