# Supplementary material for: Ionomer-Based Ion-Sensitive Field-Effect Transistor for Lithium Ion Sensing
Source: ACS Omega. 2025 Nov 26;10(48):58296–305. doi: 10.1021/acsomega.5c05196 (PMC12771037; doi:10.1021/acsomega.5c05196)
Supplement: Supplementary file 1 [file ao5c05196_si_001.pdf]

Supporting Information

Ionomer Based Ion Sensitive Field Effect Transistor for Lithium Ion Sensing

Tuluhan Olcayto Colak, [a] Mehmet Kurt, [a] Ecenaz Yaman, [a] Nurdan Demirci Sankir, [a],[b]* and Mehmet Sankir [a],[b]**

[a] Micro and Nanotechnology Graduate Program, TOBB University of Economics and Technology, Sogutozu Caddesi No 43 Sogutozu 06560 Ankara, Turkey

[b] Department of Materials Science and Nanotechnology Engineering, TOBB University of Economics and Technology, Sogutozu Caddesi No 43 Sogutozu 06560 Ankara, Turkey

Corresponding authors: **msankir@etu.edu.tr, Phone: +90 312 2924332, Fax: +90 312 292 4121 ** nsankir@etu.edu.tr, Phone: +90 312 2924331, Fax: +90 312 2924121

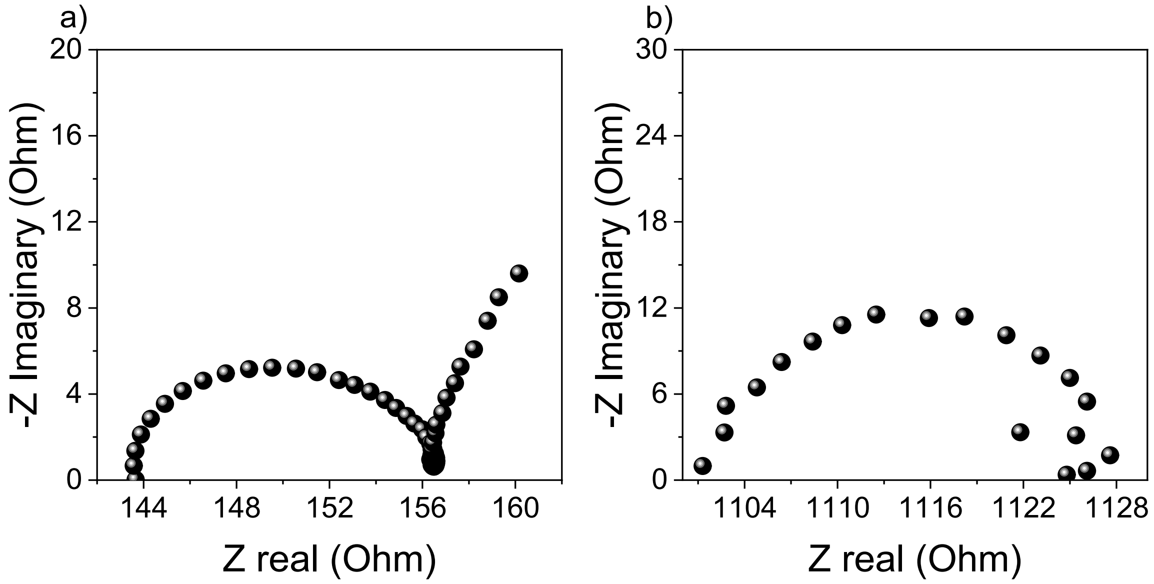

Figure S1. EIS results of the Nafion 115 membrane taken in deionized water. (a) proton form Nafion, (b) lithiated (lithium form) Nafion.

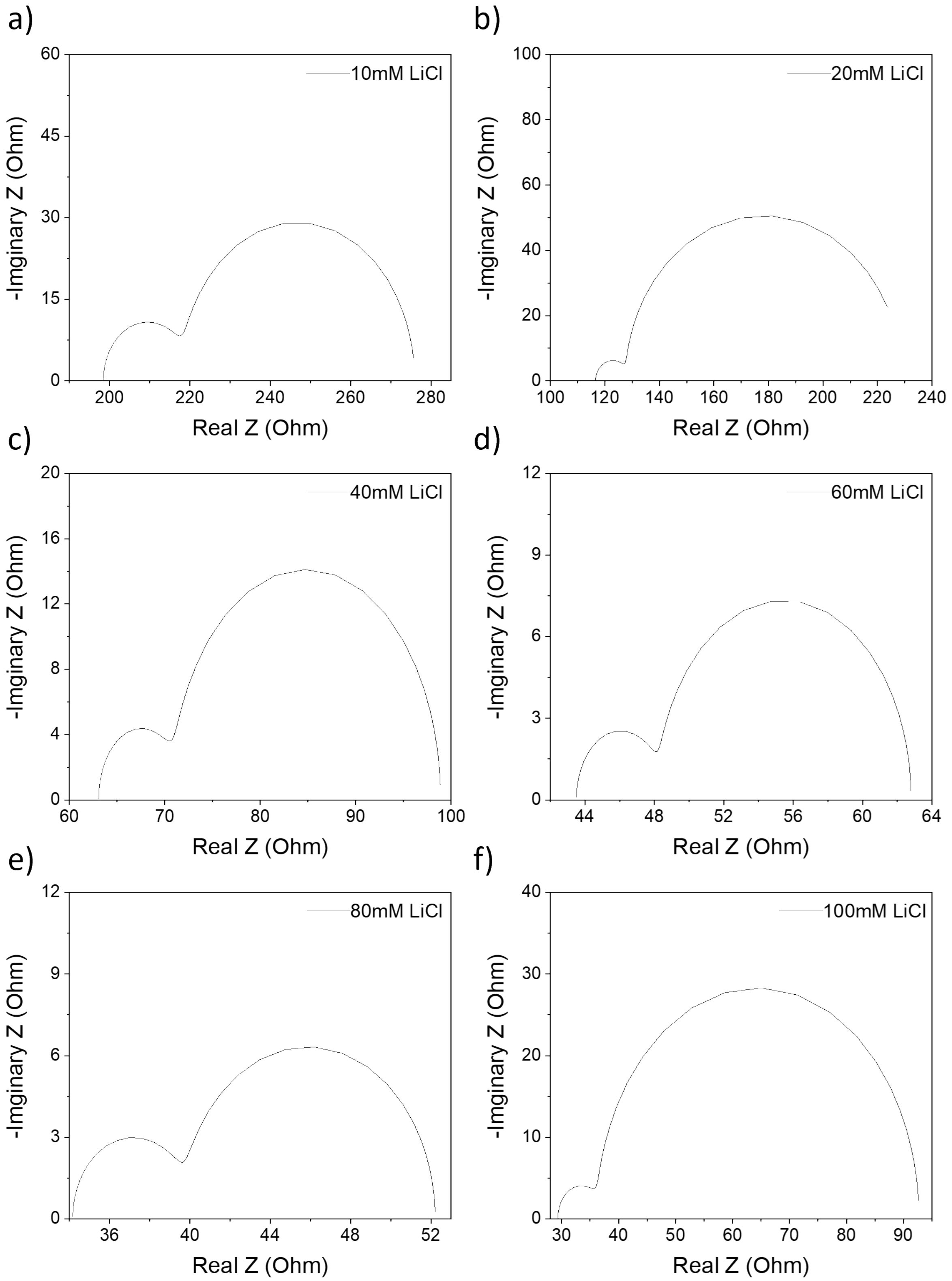

Figure S2. EIS measurements of lithiated Nafion 115 in 10mM (a), 20mM (b), 40mM (c), 60mM (d), 80mM (e), 100mM (f) lithium chloride solutions. Concentrations of were used.

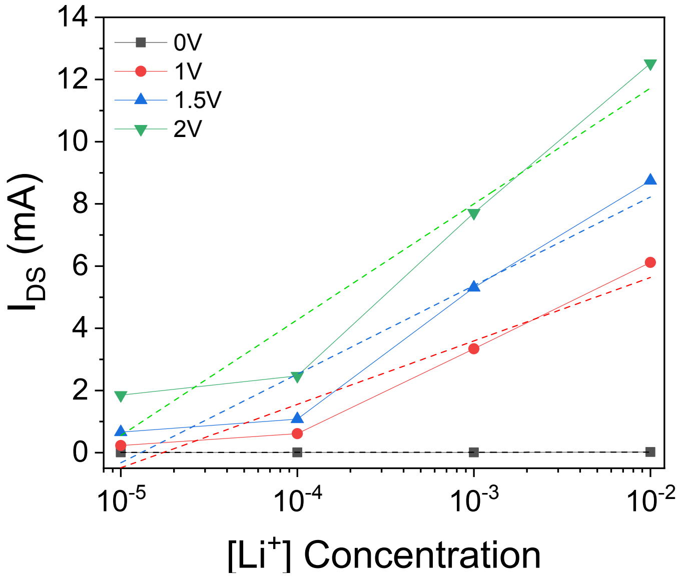

Figure S3. Calibration curves for the Nafion-gated ISFET device.

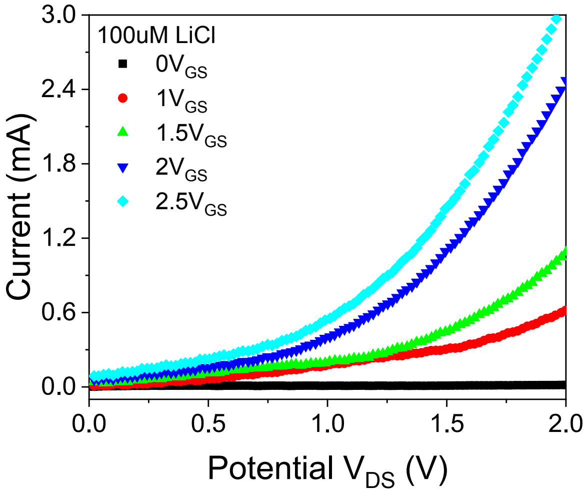

Figure S4. I-V graph of the ISFET devices operated at 100 μM LiCl.

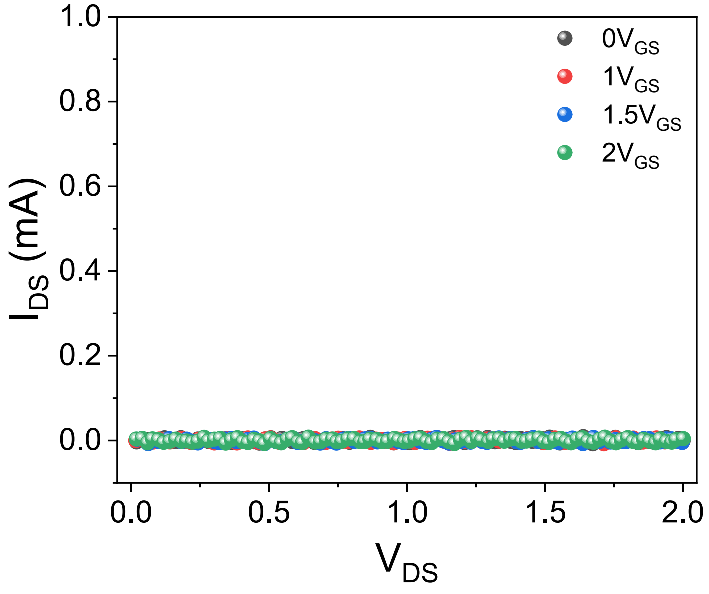

Figure S5. Linear potential sweep of ISFET prepared with PTFE taken for 1mM LiCl solution.

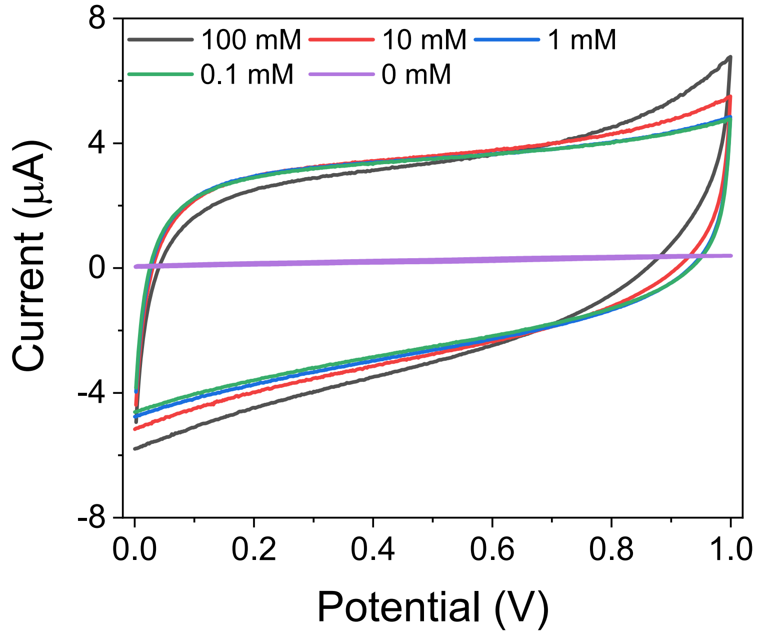

Figure S6. Cyclic voltammetry results for various concentrations taken with 0.1 V s^{-1} scan rate.

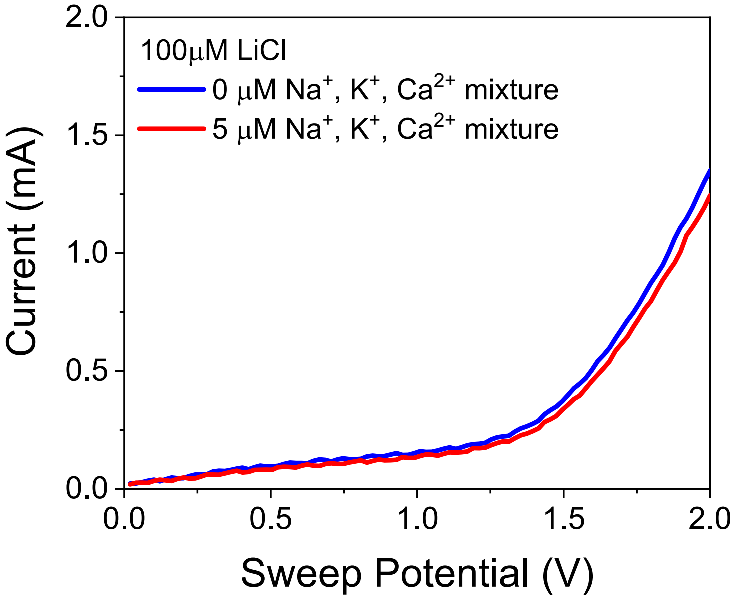

Figure S7. Linear potential sweep of ISFET prepared Nafion 115 measured using solutions of 100uM LiCl and 100uM LiCl with 5μM mixture of equimolar Na, K, and Ca cations.

Table S1. Examples of lithium-ion sensors in the literature. Highlighting variations in sensitive substances and their corresponding working range regarding molarity. The ISFET device utilizes Nafion 115 as the gate material.

**Device**	**Method**	**Working Range (mol.L^{-1})**	**Reference**
Alkyl thiol substituted copillar[4+1] arene/AuE	Capacitance by CV	10^{-2}	1
Triethylene glycolmonomethylether end-grafted carbosiloxane dendrimer (III) membrane	Chronopotentiometry	2.5×10^{-5} - 10^{-1}	2
λ-MnO_2-based graphite–epoxy	Chronopotentiometry	10^{-6} - 3.3×10^{-2}	3
Spinel $Li_{1.05}M_{0.02}Al_{1.98}O_4$ based sensor	Chronopotentiometry	10^{-5}	4
Triazabicyclo derivative monolayers/AuE	CV peak current using $[Ru(NH3)_6]Cl_3$ as redox probe	$2.5x10^{-2}$	5
DB-14-C-4/PU thin film	CV peak potential	$5x10^{-4}$ - 10^{-3}<br>$1.5x10^{-3}$ - $2.5x10^{-3}$	6
DB-14-C-4/ferrocene SEM/GCE	CV peak potential	10^{-5} - 10^{-2}	7
DB-14-C-4/11-MUA monolayer/SPAuE	DPV current signal using $[Ru(NH_3)_6]Cl_3$ as redox probe	10^{-4} - $2.5x10^{-3}$	8
Fiber-Optic Fluorescence Sensor	Emission spectroscopy	10^{-6} - 10^{-2}	9
Cotton Thread-based optical sensor with 6,6'-dibenzyl-14-crown-4 ionophore	Flame photometry	$8.8x10^{-4}$ - 0.95	10
$LiMn_2O_4$/GCE $LiMn_2O_4$/SPE	Galvanostatic step followed by LSV	$5x10^{-5}$ - $5x10^{-3}$	11
BC12 C4-NH_2/PET membrane	I–E Curve based on Poisson and Nernst-Plank Equations	10^{-5} - 10^{-1}	12
DB-14-C-4 at o-nitro phenyl ether/ water interface	Pulse amperometric current	$2x10^{-4}$ - $2x10^{-3}$	13
Dipyridophenazine derivative ligand structure	UV-Vis	$1.1x10^{-1}$	14

Table S2. Sensitivity values calculated for the device.

Sensitivity ($\partial I_D/\partial$-log[Li^+])				
Concentration difference	V_{GS} (V)			
	0	1	1.5	2
$9x10^{-3}$	0.01	2.78	3.44	4.81
$9x10^{-4}$	0	2.73	4.23	5.24
$9x10^{-5}$	0	0.38	0.42	0.62
$9x10^{-6}$	0	0.01	0.06	0.05
$9x10^{-7}$	0	0.01	0.05	0.17

Modelling Parameters

Table S3. Basic simulation parameters that were used for ISFET device simulations.

Parameters For Isfet Simulation		
Parameters	Values	Unit
Diffusion coefficient, cation	10^{-9}	m^2/s
Diffusion coefficient, anion	10^{-9}	m^2/s
Stern layer thickness	0.19479	F/m^2
Temperature	298.15	K

Table S4. Simulation parameters that were used for LiCl electrolyte used in the device.

Parameters For LiCl		
Parameters	Values	Unit
Relative permittivity	70	1
Resistivity	18.2	Ω·m
Density	2.07	g/cm^3

Table S5. Simulation parameters that were used for ZnO layer in the device.

Parameters For ZnO		
Parameters	Values	Unit
Relative permittivity	8,3	1
Electron lifetime, SRH	1.5[us]	s
Hole lifetime, SRH	1.5[us]	s
Band gap	3.37[V]	V
Electron affinity	4.49[V]	V
Effective density of states, valence band	$(T/300[K])^{(3/2)}*1.04*10^{19}[cm^{-3}]$	$1/m^3$
Effective density of states, conduction band	$(T/300[K])^{(3/2)}*2.8*10^{19}[cm^{-3}]$	$1/m^3$
Electron mobility	$180[cm^2/(V.s)]$	$m^2/(V·s)$
Hole mobility	$20[cm^2/(V.s)]$	$m^2/(V·s)$
Thermal conductivity	60[W/(m.K)]	W/(m·K)
Density	$5676[kg/m^3]$	kg/m^3

Table S6. Simulation parameters that were used for the lithiated Nafion 115 cation exchange membrane in the device.

Parameters For Nafion 115		
Parameters	Values	
Thermal conductivity	0.22	W/(m·K)
Electrolyte conductivity	0.073	S/m

References

(1) Kothur, R. R.; Flavia, F.; Gennaro, D.; Ludovic, D.; Wafa, A.; Bhavik Anil, P.; Gareth W. V., C.; Ian A., G.; Dipak K., S.; Sergey V., M.; and Cragg, P. J. Synthesis and Applications of Copillar[5]Arene Dithiols. *Supramol. Chem.* **2016**, *28* (5–6), 436–443. https://doi.org/10.1080/10610278.2015.1111375.

(2) Gupta, V. K.; Chandra, S.; Agarwal, S.; Lang, H. Lithium-Selective Potentiometric Sensor Based on a Second Generation Carbosiloxane Dendrimer. *Sensors Actuators B Chem.* **2005**, *107* (2), 762–767. https://doi.org/https://doi.org/10.1016/j.snb.2004.12.015.

(3) de S. Teixeira, M. F.; Fatibello-Filho, O.; Ferracin, L. C.; Rocha-Filho, R. C.; Bocchi, N. A λ-MnO2-Based Graphite–Epoxy Electrode as Lithium Ion Sensor. *Sensors Actuators B Chem.* **2000**, *67* (1), 96–100. https://doi.org/https://doi.org/10.1016/S0925-4005(00)00389-0.

(4) Freitas, B. H.; Amaral, F. A.; Bocchi, N.; Teixeira, M. F. S. Study of the Potentiometric Response of the Doped Spinel Li1.05Al0.02Mn1.98O4 for the Optimization of a Selective Lithium Ion Sensor. *Electrochim. Acta* **2010**, *55* (20), 5659–5664. https://doi.org/10.1016/j.electacta.2010.04.104.

(5) Wanichacheva, N.; Soto, E. R.; Lambert, C. R.; McGimpsey, W. G. Surface-Based Lithium Ion Sensor: An Electrode Derivatized with a Self-Assembled Monolayer. *Anal. Chem.* **2006**, *78* (20), 7132–7137. https://doi.org/10.1021/ac0603429.

(6) Cuartero, M.; Crespo, G. A.; Bakker, E. Polyurethane Ionophore-Based Thin Layer Membranes for Voltammetric Ion Activity Sensing. *Anal. Chem.* **2016**, *88* (11), 5649–5654. https://doi.org/10.1021/acs.analchem.6b01085.

(7) Cuartero, M.; Chai, L.; Zhang, B.; De Marco, R.; Crespo, G. A. Ferrocene Self Assembled Monolayer as a Redox Mediator for Triggering Ion Transfer across Nanometer-Sized Membranes. *Electrochim. Acta* **2019**, *315*, 84–93. https://doi.org/https://doi.org/10.1016/j.electacta.2019.05.091.

(8) Singh, U.; Kumbhat, S. Ready to Use Electrochemical Sensor Strip for Point-of-Care Monitoring of Serum Lithium. *Electroanalysis* **2021**, *33* (2), 393–399. https://doi.org/https://doi.org/10.1002/elan.202060393.

(9) Qin, W.; Obare, S. O.; Murphy, C. J.; Angel, S. M. A Fiber-Optic Fluorescence Sensor for Lithium Ion in Acetonitrile. *Anal. Chem.* **2002**, *74* (18), 4757–4762. https://doi.org/10.1021/ac020365x.

(10) Lewińska, I.; Capitán-Vallvey, L. F.; Erenas, M. M. Thread-Based Microfluidic Sensor for Lithium Monitoring in Saliva. *Talanta* **2023**, *253*, 124094. https://doi.org/https://doi.org/10.1016/j.talanta.2022.124094.

(11) Suherman, A. L.; Rasche, B.; Godlewska, B.; Nicholas, P.; Herlihy, S.; Caiger, N.;

Cowen, P. J.; Compton, R. G. Electrochemical Detection and Quantification of Lithium Ions in Authentic Human Saliva Using LiMn2O4-Modified Electrodes. *ACS Sensors* **2019**, *4* (9), 2497–2506. https://doi.org/10.1021/acssensors.9b01176.
(12) Ali, M.; Ahmed, I.; Ramirez, P.; Nasir, S.; Mafe, S.; Niemeyer, C. M.; Ensinger, W. Lithium Ion Recognition with Nanofluidic Diodes through Host–Guest Complexation in Confined Geometries. *Anal. Chem.* **2018**, *90* (11), 6820–6826. https://doi.org/10.1021/acs.analchem.8b00902.
(13) Sawada, S.; Torii, H.; Osakai, T.; Kimoto, T. Pulse Amperometric Detection of Lithium in Artificial Serum Using a Flow Injection System with a Liquid/Liquid-Type Ion-Selective Electrode. *Anal. Chem.* **1998**, *70* (20), 4286–4290. https://doi.org/10.1021/ac9805347.
(14) Obare, S. O.; Murphy, C. J. A Two-Color Fluorescent Lithium Ion Sensor. *Inorg. Chem.* **2001**, *40* (23), 6080–6082. https://doi.org/10.1021/ic010271q.
